# Determinants of disability pension following sickness absence in French private-sector employees

**DOI:** 10.1093/eurpub/ckag094

**Published:** 2026-06-21

**Authors:** Mohamed Ali Ben Halima, Karim Aït Bouziad, Narimene Louati, Marie-Anne Cousin-Renié, William Dab

**Affiliations:** CEET, Conservatoire National des Arts et Métiers, Paris, France; MESuRS, Conservatoire National des Arts et Métiers, Paris, France; MESuRS, Conservatoire National des Arts et Métiers, Paris, France; MESuRS, Conservatoire National des Arts et Métiers, Paris, France; Malakoff Humanis, Paris, France; Malakoff Humanis, Paris, France; MESuRS, Conservatoire National des Arts et Métiers, Paris, France

**Keywords:** sickness absence, disability pension, risk factors, occupational health policy, prevention

## Abstract

Disability pension represents a major public health challenge in France, with 843 000 beneficiaries and €8.5 billion expenditure in 2022. The transition from sickness absence to disability pension remains insufficiently documented in the French context. This study identifies risk factors for disability pension among French private-sector employees to inform targeted prevention strategies. We conducted a retrospective cohort study of 228 514 employees from a complementary health insurer (2018–23), encompassing 364 762 sickness absence episodes. Risk factors for transition to disability pension were analyzed using Kaplan–Meier survival and Cox proportional hazards models stratified by absence duration, adjusting for sociodemographic, employment, organizational, and health-related factors. Of all episodes, 6404 (1.8%) progressed to disability pension. Risk was particularly high among individuals aged ≥50 years and among those in higher-risk socio-professional groups, notably executives and individuals belonging to the “Other” category (including farmers, merchants, and business owners), compared with employees. Increased risk was also observed among workers in the lowest income quartile and among employees in large companies. Long-term sickness absence (>180 days) was associated with a higher risk of disability pension. Therapeutic part-time work reduced risk only for short absences. Most risk factors lost predictive power beyond 12 months, with declining effect sizes over time. This study identifies a critical 12-month intervention window. Early interventions should prioritize therapeutic part-time work and strengthen targeted support for older workers, executives, and large company employees to reduce disability pension transitions and curb rising costs.

## Introduction

In most Western countries, the number of people outside the labor market due to poor health is increasing among older individuals [1–3].

Workers increasingly shift between unemployment, sickness absence or disability pension (DP) before reaching retirement age [2, 4–6]. DP is defined as income replacement for individuals whose work capacity is permanently impaired due to health conditions [4, 5, 7, 8].

In France, DP is generally granted following long-term sickness absence, which can last up to 3 years. With 843 000 beneficiaries and total expenditure of €8.5 billion for DP in 2022 [9], DP represents a significant public health and economic challenge. The 2023 pension reform, which raised the legal retirement age from 62 to 64 years, may increase pressure on older workers to remain in employment longer, potentially exacerbating this issue as workers with declining health seek alternative exit routes from employment [10]. While international research has identified various DP predictors, studies examining these mechanisms in the specific context of the French social protection system characterized by long sickness-absence duration and heterogeneous complementary insurance coverage remain limited. Understanding risk factors for DP transition is crucial for early identification of at-risk populations and for designing effective prevention strategies.

In this context, research has identified four types of determinants that explain the risk of DP transition after sickness absence: sociodemographic factors, work-related factors, the frequency and duration of sickness absences, and health-related factors. Regarding sociodemographic factors, DP primarily affects older people [11, 12], though younger populations can also be exposed [2, 13, 14]. Results vary by sex, with some studies showing higher risk among men [15, 16] and others among women [13, 17]. Additional sociodemographic risk factors include low income, low educational level, or foreign origin [11, 18]. Having young children and being married are protective factors [11].

Concerning work-related factors, socio-professional categories play a role, with blue-collar workers typically having higher risk than white-collar workers [12, 16, 19]. Poor working conditions increase this risk [18, 20–22], and psychosocial work environment factors—such as high job strain, low decision latitude, and limited social support—have been consistently associated with an increased risk of DP, particularly for mental disorders [20–22]. Part-time work compared to full-time employment [23, 24], and presenteeism, working while ill, is a predictor of DP [25–27]. The impact of company size and sectors on DP risk remains less studied.

Regarding sickness absence patterns, long-term sickness absences are the main predictors of DP [7, 28, 29]. A Danish study showed that frequent but short absences double the risk of receiving DP [28]. In terms of health-related factors, musculoskeletal disorders and mental health problems are the two main conditions related to DP [7, 15]. Additionally, people with frequent use of medical care, such as consultations, hospitalizations and surgical interventions, have an increased risk of receiving DP [7, 29].

However, despite extensive international evidence, important gaps remain. Most existing studies are based on Nordic administrative registers with diagnostic information, limiting comparability with countries such as France where diagnostic data are not available. In addition, little research has examined how risk factors evolve over time during the sickness-absence spell, although temporal dynamics may influence transitions to disability. Finally, the institutional specificities of the French system particularly its long sickness-absence duration and complementary insurance arrangements have received limited attention.

This study aims to identify risk factors for DP among French private sector employees while examining how these associations vary over the sickness-absence trajectory. By integrating a temporal perspective, the study seeks to inform early prevention strategies and contribute to evidence-based policymaking.

## Methods

### The French sickness absence and DP system

In France, the DP system compensates for income loss due to non-work-related illness or accident [9, 30]. An employee is considered disabled if they have lost at least two-thirds of their work capacity before reaching retirement age [9, 30, 31], with the degree of incapacity assessed by social insurance physicians. France distinguishes three disability levels: partial, total, and severe incapacity (categories 1–3) [31].

During sickness absence, the Social Security system provides daily allowances equivalent to 50% of basic gross daily salary, capped at 1.8 times the minimum wage, for a maximum duration of 1095 days (3 years) [30, 31]. These allowances are typically supplemented by employers and complementary insurance schemes to ensure adequate income replacement [9].

### Data sources

The study is based on matched data from two databases of a major French social protection group, Malakoff Humanis. This insurance group provides coverage to nearly 10 million employees across ∼470 000 companies in France. Employees in these databases are covered by collective insurance contracts subscribed by employers, which include supplementary insurance covering sickness, disability and death risks. The first database contains detailed information on compensated preventive benefits, including exact sickness absence dates and transition to DP (2018–23). The second database comprises administrative data from the French Nominative Social Declaration (DSN, 2018–23), providing monthly information on the professional situation and individual characteristics of insured persons, including employee and company characteristics.

For each sickness absence episode recorded in the prevention database, corresponding individual and company data from the DSN were retrieved and matched using employee identifiers and the temporal coordinates (month and year) of absence initiation. Additionally, two lagged indicators reflecting prior sickness absence history were calculated for each sickness absence episode: the total duration of previous sickness absences in year *t*–1 and year *t*–2. These variables were introduced to capture prior work disability trajectories while limiting simultaneity bias with the current sickness absence episode. They should not be interpreted as direct measures of underlying health status, as work disability reflects an institutional evaluation of functional work capacity and labor-market participation. The final sample consists of 364 762 sickness absence episodes, representing 228 514 individuals, of whom 6404 transitioned to DP between 2018 and 2023.

### Variables

#### Sociodemographic variables

We included sex (female/male), age (categorized as <30, 30–39, 40–49, and >50 years), and income based on monthly salary quartiles: Q1 (<€1590), Q2 (€1591–2010), Q3 (€2011–2770), and Q4 (>€2770).

#### Employment characteristics

These comprised socio-professional category (executives and higher intellectual professions, intermediate professions, manual workers, employees, farmers/merchants/business owners), contract type (fixed-term contracts CDD, permanent contracts CDI, other) and working time (full-time/part-time). Socio-professional categories were defined according to the French Professions and Socio-Professional Categories (PCS—2020) classification. A detailed description of the professions included in each category (Table S1).

#### Organizational factors

Company-level variables included size (<250, 250–999, 1000–2499, 2500–4999, >5000 employees), economic sector (transport/energy/telecommunications, agriculture, industry, services, construction, commerce, health/social work), and geographic region (Île-de-France, Northwest, Northeast, Southwest, Southeast, overseas territories).

#### Work disability variables

To capture prior work disability trajectories while limiting simultaneity endogeneity bias with the current sickness absence episode, we introduced lagged variables representing the cumulative duration of sickness absences in years *t*–1 and *t*–2 (categorized as: none, <30, 30–180, >180 days). These cut-offs were chosen for pragmatic and analytical reasons to ensure analytical comparability and to distinguish short-term, intermediate, and long-term sickness-absence patterns commonly used in register-based research, rather than to reflect formal work-ability assessments. In the French disability insurance system, no specific administrative or clinical evaluation of work capacity is systematically tied to these duration thresholds. These lagged variables should be interpreted as indicators of prior sickness-absence exposure and work-disability dynamics rather than as direct measures of underlying health status. The therapeutic part-time work (TPT) allows employees to resume work with reduced working hours while receiving partial compensation from health insurance.

Table 1 presents the descriptive statistics of these variables and their association with DP.

**Table 1.** Descriptive characteristics of the study population according to DP status during follow-up.

Variable	Sample size *N* = 364 762	%	Disability rate *N* = 6404	***P*-value** ^a^
**Sociodemographic**				
Sex				<.001
Female	243 952	67%	1.4%	
Male	120 810	33%	2.4%	
Age (years)				<.001
<30	58 902	16%	0.2%	
30–39	99 264	27%	0.7%	
40–49	92 068	25%	1.8%	
≥50	114 528	31%	3.5%	
Income quartiles				<.001
Q1 (<€1590)	133 252	37%	1.6%	
Q2 (€1591–€2010)	66 263	18%	1.3%	
Q3 (€2011–€2770)	95 677	26%	1.9%	
Q4 (>€2770)	69 570	19%	2.3%	
**Employment**				
Socio-professional category				<.001
Executives	35 080	9.6%	2.8%	
Intermediate professions	92 577	25%	1.7%	
Manual workers	71 175	20%	2.6%	
Employees	165 019	45%	1.2%	
Other^b^	911	0.2%	2.1%	
Contract type				<.001
Fixed-term	16 644	4.6%	0.4%	
Permanent	344 111	94%	1.8%	
Other	4007	1.1%	0.5%	
Working time				<.001
Full-time	282 025	77%	1.8%	
Part-time	82 737	23%	1.4%	
**Organizational**				
Company size				<.001
<250	77 006	21%	1.1%	
250–999	94 586	26%	0.9%	
1000–2499	60 546	17%	1.2%	
2500–4999	36 875	10%	1.6%	
≥5000	95 749	26%	3.5%	
Economic sector				<.001
Transport/Energy/Telecommunications	19 709	5.4%	2.1%	
Agriculture	1669	0.5%	2.3%	
Industry	52 666	14%	3.6%	
Services	116 017	32%	1.8%	
Construction	4424	1.2%	2.3%	
Commerce	33 884	9.3%	1.9%	
Health/Social	136 393	37%	0.9%	
Region				<.001
Ile-de-France	55 289	15%	2.1%	
Northwest	85 864	24%	1.3%	
Northeast	93 641	26%	1.6%	
Southwest	53 641	15%	1.8%	
Southeast	73 571	20%	2.2%	
Overseas	2756	0.8%	2.5%	
**Work disability**				
TPT work				<.001
Yes	13 929	3.8%	2.7%	
No	350 833	96%	1.7%	
Previous sickness absence (*t*–1)				<.001
None	269 689	74%	2.1%	
<30 days	52 856	14%	0.2%	
30–180 days	36 247	9.9%	0.9%	
>180 days	5970	1.6%	3.3%	
Previous sickness absence (*t*–2)				<.001
None	294 867	81%	2.0%	
<30 days	37 612	10%	0.3%	
30–180 days	27 147	7.4%	0.9%	
>180 days	5136	1.4%	3.4%	

a Pearson’s chi-squared test.

b Farmers; merchants; business owners.

Source: Administrative data from Malakoff Humanis (DSN and supplementary insurance). 2018–23.

### Statistical analysis

We estimated the survival function for transition to DP using Kaplan–Meier methods. Cox proportional hazards models were used to examine factors associated with DP transitions.

The proportional-hazards assumption was tested through Schoenfeld residuals. We estimated a global model including all episodes. Stratified models by sickness-absence duration to capture temporal heterogeneity: <3, 4–12, 13–24, and 25–36 months.

Multivariate adjusted hazard ratios (HR) with 95% confidence intervals (CI) were computed.

Statistical significance was set at *P* < .05. All analyses were performed using RStudio.

## Results

### Descriptive statistics

The average duration of sickness absences leading to DP was five times longer than regular absences (504 days vs. 108 days), suggesting that DP typically occurs after prolonged and severe health deterioration (Table S2).

Men showed higher disability rates (2.4%) than women (1.4%). A strong age gradient was observed, with disability rates increasing >17-fold between the youngest (<30 years: 0.2%) and oldest age groups (≥50 years: 3.5%). Among socio-professional categories, executives (2.8%) and manual workers (2.6%) demonstrated the highest disability rates. Large companies (≥5000 employees) showed more than triple the disability rate of medium-sized companies (0.9%). Sector analysis revealed the highest transition rate in industry (3.6%), followed by construction and agriculture (both 2.3%). Health/social sectors showed the lowest rates (0.9%). Previous health patterns were also predictive: long absences (>180 days) in previous years were associated with DP rates above 3.3%. These descriptive differences underscore substantial heterogeneity across demographic, occupational and organizational groups.

### Survival analysis

**Figure 1.**
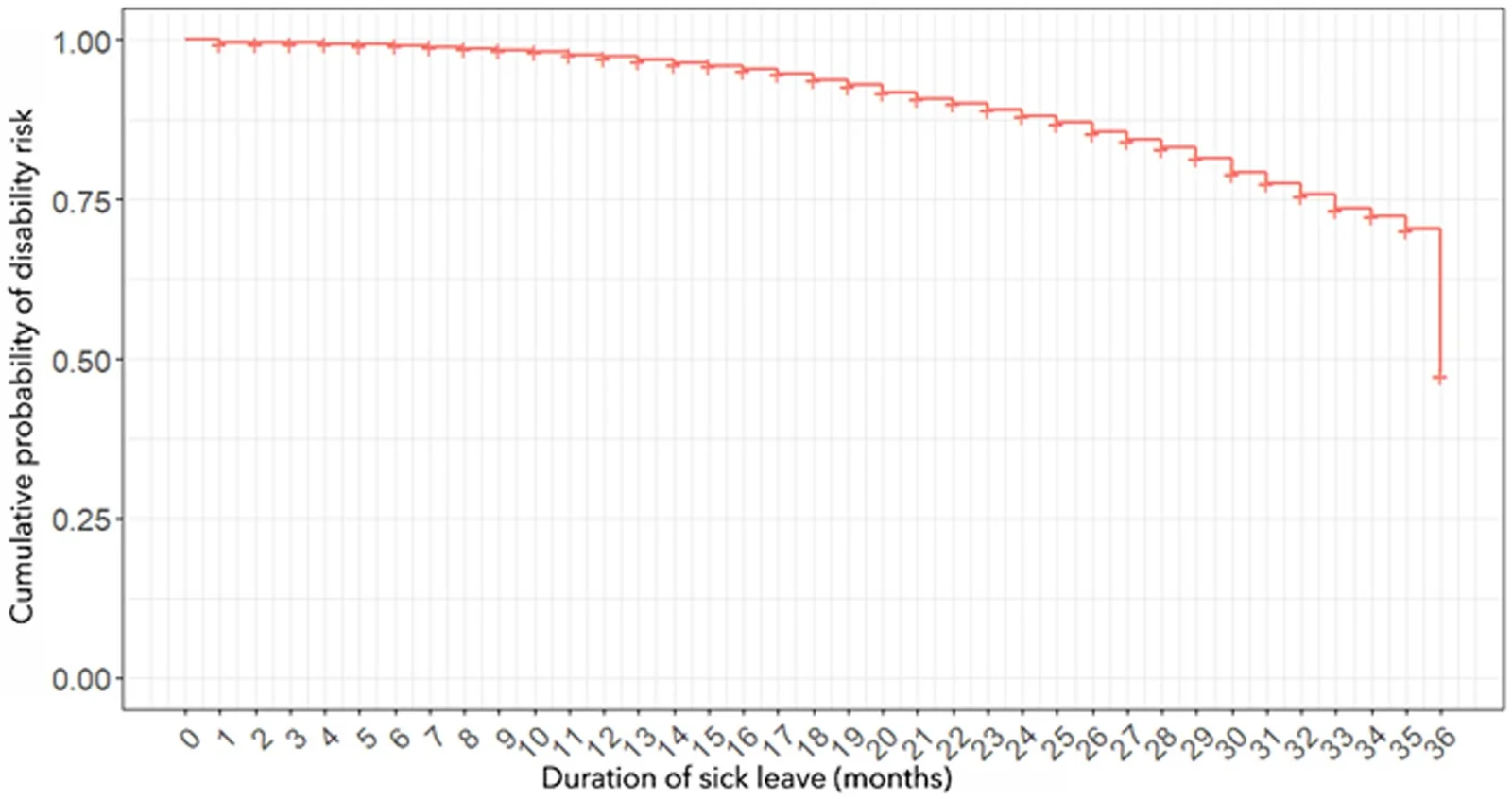
Kaplan–Meier survival function: risk of transition to disability pension by sickness absence duration in the entire sample (2018–23). Source: Administrative data from Malakoff Humanis (DSN and supplementary insurance). 2018–2023.

Kaplan–Meier analysis (Fig. 1) showed the probability of remaining on sickness absence was initially very high (99.6% after 1 month) but declined to 47.4% by the end of the 36-month legal period. Thus, more than half of workers exited sickness absence before reaching the 3-year maximum, either through return to work, transition to DP, unemployment or other routes.

Sex-stratified curves revealed similar trajectories until ∼24 months (Fig. S1). In contrast, age-stratified analysis displayed a pronounced gradient: employees ≥50 years transitioned more rapidly to DP (∼60% by 36 months), while 75% of employees <30 years remained on sickness absence at the same point (Fig. S2). These patterns confirm the dominant influence of age on DP trajectories.

### Risk factors for DP: global model

Table 2 presents the multivariable Cox model results. Among sociodemographic factors, age was the strongest determinant, the risk increasing significantly (*P* < .001) with each age category compared to employees under 30 years, particularly for those over 50 years (HR = 7.66). Income showed an inverse association, with Q4 workers having 34% lower risk (HR = 0.66) than Q1. Sex was not significantly associated with DP after adjustment.

**Table 2.** Cox proportional hazards model for disability risk (Global model).

Variable	**HR** ^a^	**95% CI** ^a^	***P*-value** ^b^
**Sociodemographic**			
Sex			
Female	1		
Male	1.01	0.95–1.07	.8
Age (years)			
<30	1		
30–39	2.60	2.10–3.23	<.001
40–49	4.75	3.86–5.85	<.001
≥50	7.66	6.24–9.39	<.001
Income quartiles			
Q1 (<€1 590)	1		
Q2 (€1591–€2010)	0.81	0.75–0.88	<.001
Q3 (€2011–€2770)	0.94	0.88–1.01	.088
Q4 (>€2770)	0.66	0.61–0.71	<.001
**Employment**			
Socio-professional category			
Employees	1		
Executives	1.44	1.31–1.58	<.001
Intermediate professions	1.20	1.12–1.29	<.001
Manual workers	1.04	0.96–1.13	.3
Other^c^	1.61	1.01–2.56	.045
Contract type			
Fixed-term	1		
Permanent	1.88	1.47–2.41	<.001
Other	1.25	0.75–2.08	.4
Working time			
Full-time	1		
Part-time	0.97	0.91–1.05	.5
**Organizational**			
Company size			
<250	1		
250–999	1.29	1.17–1.42	<.001
1000–2499	1.71	1.55–1.90	<.001
2500–4999	1.87	1.68–2.08	<.001
≥5000	2.57	2.37–2.79	<.001
Economic sector			
Industry	1		
Transport/Energy/ Telecommunications	0.71	0.64–0.79	<.001
Agriculture	1.59	1.14–2.20	.006
Services	1.03	0.95–1.11	.4
Construction	0.89	0.73–1.09	.3
Commerce	0.85	0.77–0.94	.001
Health/Social	0.79	0.72–0.86	<.001
Region			
Ile-de-France	1		
Northwest	0.87	0.80–0.95	.002
Northeast	1.05	0.97–1.14	.2
Southwest	1.30	1.19–1.42	<.001
Southeast	1.35	1.25–1.46	<.001
Overseas	1.65	1.29–2.10	<.001
**Work disability**			
Therapeutic part-time work			
No	1		
Yes	1.06	0.96–1.18	.3
Previous sickness absence (t–1)			
None	1		
<30 days	0.29	0.24–0.36	<.001
30–180 days	0.80	0.71–0.90	<.001
>180 days	1.26	1.08–1.48	.004
Previous sickness absence (t–2)			
None	1		
<30 days	0.41	0.34–0.51	<.001
30–180 days	0.77	0.68–0.88	<.001
>180 days	1.08	0.92–1.28	.3

a HR, hazard ratio; CI, confidence interval.

b Pearson’s chi-squared test.

c Farmers; merchants; business owners.

Source: Administrative data from Malakoff Humanis (DSN and supplementary insurance). 2018–23.

A notable finding among employment factors was the relationship between socio-professional categories and disability risk. Using employees as the reference category, executives exhibited a higher risk of DP (HR = 1.44; 95% CI, 1.31–1.58). Intermediate professions also showed increased risk (HR = 1.20; 95% CI, 1.12–1.29), whereas manual workers did not differ significantly from employees (HR = 1.04; 95% CI, 0.96–1.13). The “Other” category (farmers, merchants, and business owners) showed a higher hazard ratio (HR = 1.61; 95% CI, 1.01–2.56). Workers with permanent contracts had higher risk (HR = 1.88) than those with fixed-term contracts. Working time (full-time vs. part-time) was not significant.

Among organizational factors, employees in large companies showed significantly higher DP risk than those in smaller companies, with risk increasing progressively by company size (HR = 2.57 for companies ≥5000 employees compared to companies with <250 employees). Agriculture presented the highest sectoral risk (HR = 1.59). Transport/energy/telecommunications (HR = 0.71) and health/social work (HR = 0.79) were protective relative to industry. Regional inequalities were marked: compared to Île-de-France, risk was lower in the Northwest but significantly higher in the Southwest, Southeast and overseas territories.

Regarding health-related factors, previous sickness absence history showed distinct patterns. Employees with Short (<30 days) and moderate (30–180 days) prior absences in *t*–1 and *t*–2 were strongly protective (HR = 0.29–0.80). Long prior absence (>180 days) increased risk in *t*–1 (HR = 1.26). TPT was not significant in the global model.

### Risk factors for transition to DP stratified by sickness absence duration

Table 3 shows substantial heterogeneity across duration categories. We conducted several estimations into four sickness absence duration intervals: <3, 4–12, 13–24, and 25–36 months.

**Table 3.** Cox proportional hazards models stratified by sickness absence duration (2018–23).

Variable	<3 months			4–12 months			13–24 months			25–36 months		
	**HR** ^a^	**95% CI** ^a^	*P*-value	**HR** ^a^	**95% CI** ^a^	*P*-value	HR^a^	**95% CI** ^a^	*P*-value	**HR** ^a^	**95% CI** ^a^	***P*-value** ^b^
**Sociodemographic**												
Sex												
Female	1			1			1			1		
Male	1.03	0.91–1.16	.6	0.93	0.81–1.06	.3	1.10	0.98–1.23	.12	0.94	0.85–1.04	.2
Age (years)												
<30	1			1			1			1		
30–39	5.19	2.98–9.02	<.001	3.97	2.07–7.61	<.001	1.91	1.18–3.08	.008	1.31	0.97–1.76	.074
40–49	19.4	11.4–33.1	<.001	8.09	4.29–15.2	<.001	3.39	2.14–5.38	<.001	1.61	1.21–2.13	<.001
≥50	59.4	35.0–101	<.001	15.9	8.49–29.7	<.001	5.20	3.30–8.19	<.001	1.71	1.29–2.26	<.001
Income quartiles												
Q4 (>€2770)	1			1			1			1		
Q3(€2011–2770)	2.08	1.78–2.42	<.001	1.62	1.35–1.94	<.001	1.27	1.09–1.48	.002	1.07	0.94–1.22	.3
Q2(€1591–2010)	1.02	0.82–1.27	.9	1.50	1.20–1.87	<.001	1.43	1.20–1.70	<.001	0.98	0.84–1.14	.8
Q1 (<€1590)	2.39	2.02–2.83	<.001	1.93	1.59–2.33	<.001	1.33	1.13–1.56	<.001	1.00	0.87–1.15	>.9
**Employment**												
Socio-professional category												
Employees	1			1			1			1		
Executives	1.65	1.38–1.99	<.001	1.42	1.13–1.79	.003	1.42	1.18–1.71	<.001	1.29	1.11–1.51	.001
Intermediate professions	0.99	0.86–1.15	>.9	1.29	1.09–1.54	.003	1.23	1.06–1.42	.005	1.25	1.10–1.41	<.001
Manual workers	0.98	0.83–1.17	.9	1.21	1.00–1.46	.046	1.07	0.91–1.26	.4	0.95	0.82–1.09	.5
Other^c^	1.39	0.57–3.40	.5	2.14	0.84–5.44	.11	1.15	0.36–3.66	.8	1.30	0.57–2.96	.5
Contract type												
Fixed-term	1			1			1			1		
Permanent	6.50	3.74–11.3	<.001	1.49	0.84–2.63	.2	1.23	0.78–1.94	.4	1.56	1.01–2.40	.044
Other	1.26	0.35–4.49	.7	2.16	0.88–5.29	.093	1.62	0.59–4.44	.3	0.66	0.19–2.28	.5
Working time												
Full-time	1			1			1					
Part-time	1.58	1.39–1.80	<.001	0.79	0.67–0.94	.007	0.80	0.69–0.92	.002	0.75	0.65–0.86	<.001
**Organizational**												
Company size												
<250	1			1			1			1		
250–999	1.53	1.24–1.90	<.001	1.21	0.96–1.54	.11	1.33	1.10–1.60	.003	1.21	1.03–1.43	.018
1000–2499	2.49	1.98–3.13	<.001	1.77	1.39–2.26	<.001	1.75	1.43–2.14	<.001	1.40	1.17–1.67	<.001
2500–4999	3.82	3.03–4.81	<.001	2.34	1.82–3.01	<.001	1.70	1.37–2.11	<.001	1.38	1.15–1.67	<.001
≥5000	11.5	9.59–13.7	<.001	2.44	2.00–2.97	<.001	2.17	1.85–2.54	<.001	1.58	1.38–1.81	<.001
Economic sector												
Industry	1			1			1			1		
Transport/Energy	0.63	0.51–0.79	<.001	0.66	0.51–0.87	.003	0.55	0.43–0.70	<.001	0.79	0.65–0.96	.017
Agriculture	1.98	1.16–3.36	.012	1.65	0.68–4.02	.3	1.68	0.89–3.18	.11	0.92	0.45–1.86	.8
Services	0.68	0.58–0.80	<.001	0.95	0.79–1.14	.6	0.95	0.82–1.10	.5	1.24	1.08–1.42	.002
Construction	0.33	0.20–0.55	<.001	0.82	0.46–1.47	.5	1.02	0.69–1.50	>.9	1.29	0.95–1.76	.10
Commerce	0.41	0.33–0.51	<.001	0.81	0.65–1.02	.079	0.84	0.70–1.02	.074	0.98	0.82–1.16	.8
Health/Social	0.16	0.13–0.19	<.001	0.96	0.78–1.17	.7	0.98	0.83–1.16	.8	1.12	0.96–1.32	.15
Region												
Ile-de-France	1			1			1			1		
Northwest	0.60	0.50–0.71	<.001	0.68	0.55–0.84	<.001	0.95	0.79–1.13	.5	1.02	0.88–1.18	.8
Northeast	0.83	0.70–0.97	.020	0.75	0.62–0.92	.005	1.02	0.87–1.21	.8	1.26	1.09–1.44	.001
Southwest	0.70	0.59–0.84	<.001	1.12	0.92–1.37	.3	1.32	1.11–1.58	.002	1.49	1.27–1.73	<.001
Southeast	0.86	0.74–1.01	.073	1.11	0.93–1.34	.3	1.42	1.22–1.66	<.001	1.57	1.37–1.80	<.001
Overseas	1.17	0.64–2.14	.6	1.02	0.55–1.87	>.9	1.96	1.31–2.93	<.001	2.23	1.41–3.52	<.001
**Health-related**												
TPT work												
No	1			1			1			1		
Yes	0.73	0.53–1.00	.053	0.82	0.66–1.03	.083	0.95	0.79–1.15	.6	1.37	1.14–1.64	<.001
Previous absence (*t*–1)												
None	1			1			1			1		
<30 days	0.06	0.04–0.11	<.001	0.57	0.38–0.87	.008	0.68	0.47–0.98	.039	0.37	0.25–0.56	<.001
30–180 days	0.20	0.14–0.27	<.001	1.32	1.07–1.63	.011	1.21	0.99–1.49	.064	0.55	0.43–0.72	<.001
>180 days	1.28	0.94–1.74	.12	1.60	1.20–2.13	.001	1.52	1.12–2.07	.007	0.43	0.27–0.71	<.001
Previous absence (*t*–2)												
None	1			1			1			1		
<30 days	0.12	0.08–0.20	<.001	0.83	0.56–1.23	.4	0.56	0.38–0.81	.002	0.46	0.29–0.73	<.001
30–180 days	0.26	0.19–0.38	<.001	1.28	1.01–1.62	.039	0.72	0.55–0.93	.011	0.88	0.68–1.14	.3
>180 days	1.69	1.24–2.30	<.001	2.36	1.80–3.11	<.001	0.42	0.27–0.67	<.001	0.60	0.39–0.92	.019

a HR, hazard ratio; CI, confidence interval.

b Pearson’s chi-squared test.

c Farmers; merchants; business owners.

Source: Administrative data from Malakoff Humanis (DSN and supplementary insurance). 2018–23.

#### Short absences (<3 months)

Age was the dominant predictor, with extremely high risks for workers aged 40–49 (HR = 19.4) and especially ≥50 (HR = 59.4). Income inequalities were strongest during this period, as workers in Q1 had more than double the risk of those in Q4 (HR = 2.39). Compared with employees, executives showed a higher risk of DP transition (HR = 1.65; 95% CI, 1.38–1.99), whereas intermediate professions and manual workers did not differ significantly from employees. Company size had a pronounced effect, notably in firms ≥5000 employees (HR = 11.5). TPT showed a borderline protective effect (HR = 0.73), while very short absences in t–1 (<30 days) were strongly protective (HR = 0.06).

#### Medium absences (4–12 months)

Age effects declined but remained substantial, with workers ≥50 years still at high risk (HR = 15.9). Income remained significant though attenuated. Compared with employees, executives exhibited the highest risk (HR = 1.42), followed by intermediate professions (HR = 1.29) and manual workers (HR = 1.21), all showing significantly higher risks of DP transition. Company size remained a strong predictor for DP risk. Long prior absences in year t–1 (>180 days) significantly increased the risk of transition (HR = 1.60).

#### Long absences (13–24 months)

Age gradients weakened further, with HRs dropping to 5.20 for ≥50 years and 3.39 for 40–49 years. TPT no longer had a significant effect. Long prior absences (>180 days) remained associated with increased risk (HR = 1.52). Company-size effects persisted but were less pronounced than in earlier stages.

#### Very long absences (25–36 months)

Beyond 2 years of absence, most risk factors lost predictive power. Age differences became modest (HR = 1.71 for ≥50 years), and income was no longer significant. Company size continued to matter but with reduced effect. TPT became positively associated with DP (HR = 1.37), likely reflecting indication bias among the most severe cases. Long prior absences (>180 days) shifted from being a risk factor to becoming protective, consistent with a survival-of-the-fittest dynamic.

## Discussion

This study provides new evidence on the sociodemographic and organizational determinants of DP among French private-sector employees on sickness absence. Using a large administrative cohort, we identify both well-established predictors—such as older age and long previous sickness absences—and several patterns that appear distinctive to the French labor market and social protection system.

Age emerged as the strongest predictor, particularly during early phases of sickness absence, consistent with international studies showing accelerated transitions to DP in older workers. The predictive power of most factors diminished after the first 12 months of absence, indicating a window for preventive intervention. Employment conditions were also central: executives showed a significantly higher risk of DP compared with employees, a pattern that differs from findings reported in Nordic register-based studies. As detailed in Table S1, the executive category encompasses a heterogeneous set of occupations with potentially diverse working conditions, psychosocial exposures, and institutional coverage patterns. This heterogeneity should be taken into account when interpreting occupational gradients in DP risk. Similarly, individuals belonging to the “Other” socio-professional category (farmers, merchants, and business owners) also showed elevated risk estimates; however, these should be interpreted with caution due to the smaller size and heterogeneous composition of this group. These patterns may partly reflect structural features of the French disability insurance system, including differences in eligibility rules, contribution histories, and complementary insurance coverage across occupational groups.

Company size displayed a strong gradient, with employees in very large firms experiencing substantially higher DP risks, suggesting organizational influences that merit further investigation. TPT demonstrated a protective effect for short sickness absences but not for prolonged periods. This dual pattern likely reflects indication bias, as TPT is both a return-to-work tool and an accommodation for more severe health conditions. Without diagnostic data, these effects should be interpreted cautiously.

### Comparison with existing literature

The literature emphasizes that long-term sickness absences increase the risk of DP [7], often linked to chronic health problems. In our study, only absences of >180 days in the previous year significantly increased the risk, consistent with another study reporting that previous absences exceeding 100 days increased DP risk [7]. Advanced age is a major risk factor, as confirmed by several studies [11, 12]. Our results find no significant difference between sexes, also observed in other studies [7, 23].

Previous research has reported mixed findings: one study reported increased DP risk with part-time work [23], while another found lower associated risks [16]. Our findings are consistent with this heterogeneity, as the association between part-time work and DP risk varied by sickness absence duration, with higher observed risks during short absence spells (<3 months) and lower risks during longer absence episodes.

This association should be interpreted cautiously, as it may reflect selection effects, with individuals experiencing more severe health impairments being more likely to work part-time, particularly at early stages of sickness absence. Conversely, part-time work may also function as a workplace accommodation supporting continued labor-market participation among those with long-term health limitations. Given the absence of detailed clinical information in our administrative data, we cannot determine whether part-time work has a causal protective or risk-enhancing effect on DP transitions. Our study shows significantly higher risk among executives compared with employees, contrasting with most literature reporting higher risk among manual workers [8]. However, some European study demonstrates that white-collar occupational status is associated with increased mental disorder-related DP risk [32]. This French pattern may reflect structural differences in social protection: eligibility for DP requires minimum salary contributions during the 12 months preceding absence, with pension calculated on best 10 years of earnings. Moreover, supplementary insurance is mandatory for executives under the 1947 National Collective Agreement but optional for non-executives [33], potentially leading non-executives toward alternative exit routes such as unemployment benefits near retirement [5].

### Implications for policy and practice

Three public health implications arise. First, the first year of sickness absence constitutes a critical intervention window: early occupational health assessments and return-to-work strategies may prevent long-term incapacity [34]. Second, organizational characteristics, company size, economic sector and employment contracts play a substantial role and should be incorporated into workplace prevention strategies. Third, in the context of pension reforms extending working life, early identification of workers at risk of permanent disability is essential for reducing inequitable labor-market exits.

Our findings highlight key periods and worker groups that may benefit from earlier occupational health monitoring and tailored support. The first 12 months of sickness absence represent a period during which most predictors of DP show their strongest associations. This suggests that preventive actions may be most relevant when implemented early, although our study does not assess intervention effectiveness.

TPT appears to be an important mechanism associated with reduced DP risk during early sickness absence. This scheme shows the strongest protective effects for absences under 3 months, making its early use relevant for reducing DP risk and supporting progressive return to work. However, timing is critical: employees attempting early return (within 3 months) require enhanced support, while part-time work is associated with lower DP risk after 4 months of absence, emphasizing the need for tailored occupational health interventions. Given the selection bias and the complexities of the relationship, it is not possible to definitively determine whether part-time work is “protective” regarding DP. Further research is needed to better understand these effects, especially in the absence of diagnostic data.

Several populations require targeted interventions. Employees over 50 years face significantly higher risk during the initial 12 months, necessitating regular health assessments and workplace adaptations from absence onset. Employees in large companies showed particularly elevated risks early in the absence spell. These patterns indicate that occupational health surveillance may need to be adapted to worker profiles.

Prevention strategies should include absence management programs, medical monitoring and workplace improvements. Executives require specific support given their elevated risk profile. Tools like keeping-in-touch meetings and pre-return exams help support these groups [35].

Beyond individual risk profiles, sectoral contexts influence DP risk. Agriculture and industry sectors showed higher risks, suggesting that working conditions and organizational constraints may contribute to differential disability trajectories. Enhanced coordination between private insurers, public authorities and employers could ensure equitable prevention access across all professional categories.

Our results particularly emphasize the importance of employer-led interventions during the first 12 months of absence, especially in large companies and for executives, who our findings identify as high-risk groups for DP transitions.

### Strengths and limitations

This study is among the few French investigations exploring how sickness absence duration impacts DP risk considering individual and occupational factors. We utilized high-precision administrative data collected monthly, providing information on sickness absences and DP transitions. By combining DSN data with insurance claims databases, we linked sickness absence and DP information with employees’ professional characteristics during their sickness absence periods, offering comprehensive insight into career trajectories and exact employment situations at absence onset.

The study spans 2018–23 with retrospective histories, allowing us to reconstruct prior sickness absence trajectories. Despite these strengths, several limitations exist. Although our data cover 228 514 employees insured by Malakoff Humanis, the sample reflects the insurer’s portfolio of affiliated companies and may not represent all French private-sector firms in terms of sectoral and company-size distribution, which may limit generalizability. Our study faces three main data limitations: (i) agricultural and construction workers are only sparsely represented in our dataset, as these sectors are predominantly covered by alternative provident insurance schemes rather than the DSN-based system used in our study, (ii) absence of important individual-level variables like education and family status, which are linked to DP outcomes [9, 11, 13], (iii) the dataset lacks of detailed health indicators, including the medical reasons for sickness absences and the underlying chronic conditions. This absence of diagnostic data limits the ability to distinguish between heterogeneous health trajectories and restricts the development of condition-specific prevention strategies [7, 13, 29], (iv) the dataset does not include the reasons for leaving employment (e.g. unemployment or retirement), which prevents identifying alternative exit pathways and therefore limits the use of competing-risks or multi-state models, and (v) unmeasured workplace-level factors such as management practices and working conditions [18, 20, 36], and workplace accommodations [37–39], which are recognized as important determinants of disability risk and influence DP transitions. Future research should include occupational exposure data, as recent French research demonstrates their impact on health outcomes [40]. Additionally, part of the study period overlapped with the COVID-19 pandemic, which may have influenced sickness absence patterns and DP transitions. Therefore, caution is warranted when generalizing these findings.

Additionally, our results may be affected by healthy worker effect bias, where healthier individuals remain employed while those with health problems leave the workforce. We partially addressed the lack of health data by using TPT and lagged absence variables, which offer indirect health indicators, reducing potential biases.

## Conclusions

This study provides new evidence on how risk factors for DP evolve during sickness absence. By analyzing 364 762 sickness absence episodes over 6 years, we identify a critical 12-month period during which most predictors show their strongest associations, after which their predictive power declines. Age remains the most influential factor. A notable pattern specific to the French context is the higher DP risk observed among executives compared with employees, a result likely linked to structural features of the French social protection and complementary insurance system rather than to occupational status alone. TPT shows a protective association during the early stages of absence, supporting its role in facilitating gradual return-to-work. Distinct patterns in agriculture and industry also point to the need for sector-specific approaches.

The temporal concentration of risk factors suggests prioritizing preventive measures and workplace adjustments within the first year of absence. Companies should reinforce structured absence management, occupational health services should focus on early work adaptations, and insurers may need to reassess coverage mechanisms that can influence pathways toward DP. However, the absence of diagnostic information and workplace-level data limits the precision with which targeted interventions can be designed.

Future research should clarify the mechanisms driving occupational differences in DP risk, particularly the roles of insurance structure and work conditions, and should assess the effectiveness of early interventions within the 12-month window. Improving early identification and support for high-risk workers could help reduce DP transitions in France.

## Supplementary Material

ckag094_Supplementary_Data

## Data Availability

The data underlying this article were provided by Malakoff Humanis under license and cannot be shared publicly. Key points Risk factors for DP lose predictive power after 12 months of sickness absence, establishing a critical intervention window Advanced age (≥50 years) and executive status are among the strongest predictors of DP compared with employees. Therapeutic part-time work provides significant protection when implemented within the first three months of absence onset Large company employees face highest risk during the first three months, requiring early comprehensive absence management programs
