# Supplementary material for: Determinants of disability pension following sickness absence in French private-sector employees
Source: Eur J Public Health. 2026 Jun 21;36(4):ckag094. doi: 10.1093/eurpub/ckag094 (PMC13283447; doi:10.1093/eurpub/ckag094)
Supplement: ckag094_Supplementary_Data [file ckag094_supplementary_data.zip › ejph-2025-11-om-1021-File006.docx]

**Table S1:** Definition of socio-professional categories used in the study

| **Socio-professional category** | **Main professions included (PCS 2020 – level 2)** |
| --- | --- |
| **Executives** | Liberal professions; senior civil servants; professors and higher scientific professions; information, arts and entertainment professionals; administrative and commercial managers; engineers and technical managers |
| **Intermediate professions** | Primary and vocational teachers; intermediate health and social care professions; intermediate civil service occupations; administrative and commercial associate professionals; technicians; supervisory workers |
| **Employees** | Public-sector administrative employees and service staff; private-sector administrative employees; retail and sales workers; personal and household service workers |
| Manual workers | Skilled and unskilled industrial and artisanal workers; transport and logistics workers; drivers; warehouse workers; machine operators |
| Other (farmers, merchants, business owners) | Farmers and agricultural workers; craftsmen; shopkeepers; business owners |

Note: Categories are based on the French Professions and Socio-Professional Categories (PCS – 2020) classification developed by INSEE, using level-2 groupings.
