# Supplementary material for: Determinants of disability pension following sickness absence in French private-sector employees
Source: Eur J Public Health. 2026 Jun 21;36(4):ckag094. doi: 10.1093/eurpub/ckag094 (PMC13283447; doi:10.1093/eurpub/ckag094)
Supplement: ckag094_Supplementary_Data [file ckag094_supplementary_data.zip › ejph-2025-11-om-1021-File007.docx]

**Table S2:** Descriptive Statistics for Continuous Variables

| **Sample** | **Variable** | **Mean** | **P25** | **Median** | **P75** | **SD** |
| --- | --- | --- | --- | --- | --- | --- |
| Full sample | N = 364 762 |  |  |  |  |  |
|  | Sickness absence duration (days) | 115 | 9 | 37 | 131 | 190 |
|  | Age at absence onset | 43 | 34 | 43 | 53 | 11 |
| Regular sickness absences | N = 358 358 |  |  |  |  |  |
|  | Sickness absence duration (days) | 108 | 9 | 36 | 126 | 176 |
|  | Age at absence onset | 43 | 33 | 43 | 53 | 11 |
| Sickness absences with transition to disability pension | N = 6 404 |  |  |  |  |  |
|  | Sickness absence duration (days) | 504 | 101 | 492 | 873 | 396 |
|  | Age at absence onset | 51 | 47 | 53 | 57 | 11 |

Source: Administrative data from Malakoff Humanis (DSN and supplementary insurance), 2018-2023.
