# Supplementary figures and images for: Determinants of disability pension following sickness absence in French private-sector employees
Source: Eur J Public Health. 2026 Jun 21;36(4):ckag094. doi: 10.1093/eurpub/ckag094 (PMC13283447; doi:10.1093/eurpub/ckag094)

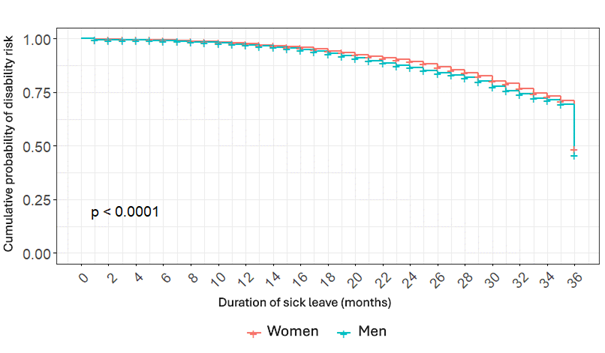

Supplement: ckag094_Supplementary_Data [file ckag094_supplementary_data.zip › ejph-2025-11-om-1021-File008.tif]

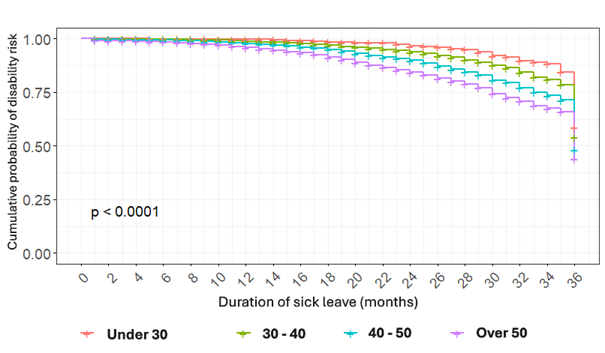

Supplement: ckag094_Supplementary_Data [file ckag094_supplementary_data.zip › ejph-2025-11-om-1021-File009.tif]
